# National survey regarding obstetricians’ perspective of obstetric emergencies in Brazil

**DOI:** 10.1016/j.clinsp.2024.100333

**Published:** 2024-02-07

**Authors:** Vitória Espindola Leite Borges, Francisco Barbosa Jr, Fábio Fernandes Neves, Maria Rita de Souza Mesquita, Elaine Christine Dantas Moisés

**Affiliations:** a Department of Gynecology and Obstetrics, Faculty of Medicine, Universidade de São Paulo, Ribeirão Preto, SP, Brazil; b Department of Medicine, Universidade Federal de São Carlos, São Carlos, SP, Brazil; c Associação de Ginecologia e Obstetrícia de São Paulo (SOGESP), São Paulo, SP, Brazil

**Keywords:** Maternal mortality, Training, Obstetric emergencies

## Abstract

**Introduction:**

The maternal mortality rate in developing countries, such as Brazil, has significantly increased since 2020. Obstetric Emergencies (OE) account for 72.5% of these deaths. A national survey was conducted in Brazil to evaluate how gynecologists and obstetricians deal with OE and identify the main difficulties regarding theoretical/practical knowledge and structural resources.

**Methods:**

An electronic questionnaire assessing resource availability, health teams, institutional protocols, and provision of OE training courses was completed by Brazilian obstetricians.

**Results:**

More than 90 % of the questionnaire respondents reported treating a pregnant and/or puerperal patient with severe morbidity and that their health network has human resources, trained professionals, and structural resources required for this type of care. However, few respondents participate in continuing education programs (36 %) or specific training for the medical team (61.41 %). The implementation rates of obstetric risk identification protocols (33.09 %), a rapid response team (46.54 %), and boxes and emergency cart assembly teams (71.68 %) were determined.

**Conclusion:**

A high Maternal Mortality Ratio (MMR) may be related to disorganized healthcare systems, low implementation of risk classification protocols for the care of severe maternal and fetal conditions, and lack of access to continued/specific training programs. The Brazilian MMR is multifactorial. According to obstetricians, Brazilian health services include care teams, essential medications, obstetric centers, and clinical analysis laboratories, though they lack systematized processes and permanent professional training for qualified care of OE.

## Introduction

Maternal Mortality (MM) is defined as the death of a woman during pregnancy or up to 42 days after delivery or termination of the pregnancy, from any cause related to or aggravated by the pregnancy, excluding deaths from external causes. The Maternal Mortality Ratio (MMR) is an important indicator of a region's development and economic-social inequality.^1^

In 2012, the World Health Organization (WHO) established sustainable development goals to solve the most urgent global problems in the political, environmental, and economic scope.^2^ Women's health was presented as a topic of great relevance, especially when discrepancies between maternal and neonatal mortality rates among developed, developing, and underdeveloped countries were evaluated.^3^

The need for the improvement of women's healthcare was emphasized in 2015 as the Global Strategy for Women's, Children's, and Adolescents’ Health was revised, establishing ambitious proposals and targets to reduce MM.^2^ The goal proposed for Brazil was to reduce the MMR from 64.4 to 30 deaths per 100,000 live births.^4^, ^5^, ^6^, ^7^ As of 2019, approximately 72.5% of cases of MM were due to hypertensive syndromes, bleeding, infections, intrapartum complications, or unsafe abortions.^1^^,^^8^ As treatments for these events have been established in national and international protocols, the proper application of these protocols would reduce the MM by 92 %.^1^^,^^9^^,^^10^

The risk classification of patients by a qualified professional plays a fundamental role in improving women's health care, as the use of a deficient triage system as the first contact between patients and health services is a barrier to welcoming, identifying, and approaching patients with severe conditions. This can be accomplished via the use of the Modified Early Obstetric Warning Score (MEOWS).^11^ Factors associated with a high MMR in Brazil must be identified so that effective protocols can be designed and implemented to reduce the MMR.

Therefore, the identification and monitoring of care provided to patients who had a serious obstetric complication during pregnancy, childbirth, or within 42 days of the termination of pregnancy and survived due to early risk identification and correct and urgent medical intervention can be used as a valuable auxiliary tool to measure the capacity of the health system to prevent and manage Obstetric Emergencies (OE). These data can also be used to identify intervention points aimed at optimizing resources and processes.^9^ To evaluate the capacity of gynecologists and obstetricians to manage OE and to identify the main difficulties of these professionals related to theoretical and practical knowledge and structural and input resources, a questionnaire regarding these topics was administered to obstetricians in Brazil.

## Materials and methods

This is an observational, cross-sectional study of the experience and opinions of physicians specialized in gynecology and obstetrics using data obtained via a single-format electronic questionnaire conducted between June 2021 and August 2022.

The electronic questionnaire included items with objective responses and allowed for subjective considerations, addressing topics related to the knowledge, experience, structure, and necessary inputs for the adequate management of OE. All professionals who answered this questionnaire provided informed consent. The electronic questionnaire was sent to the participants to characterize the care practices, structural and human resources, and operational processes of the institutions where they work.

The questionnaire was distributed to resident and professional physicians specializing in gynecology and obstetrics with an active registration in the Brazilian Federation of Gynecology and Obstetrics Association (*Federação Brasileira das Associações de Ginecologia e Obstetrícia* ‒ FEBRASGO) (n = 15,000). Questionnaires of respondents who did not provide informed consent and duplicate questionnaires from the same respondent (linked via the regional medical council (*Conselho Regional de Medicina*) number, were excluded from the study.

The following variables were analyzed: age, stage of medical residency or years as a professional, subspecialty, geographic location, type of institution (public or private, level of complexity, and back office structure), participation in obstetric care, patient screening protocol use, MEOWS use, access to an assistant team specializing in intensive care, access to an advanced life support protocol, ability and experience managing OE (bleeding, infection, and hypertension), and access to OE supplies.

**Fig. 1:**
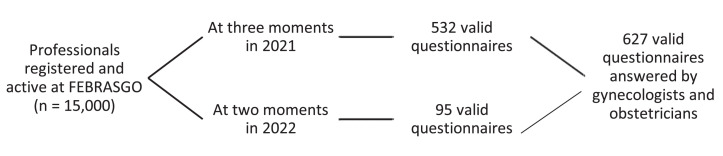
Respondent flowchart.

Invitations to complete the questionnaire were e-mailed to approximately 15,000 gynecologists and obstetricians registered in the FEBRASGO database at five moments. A total of 627 completed questionnaires were included in the final analysis (Fig. 1).

### Statistical analysis

Initially, a descriptive analysis of quantitative variables was carried out. The qualitative variables were categorized and summarized considering absolute and relative frequencies, arranging them in graphs and tables and the evaluation of variables by groups was carried out using the Chi-Square test.

For correlation analyses, the authors used the Spearman coefficient. To evaluate the power of visualizing the Spearman “*r*” coefficients, the authors considered:

• *r* greater than or equal to 0.30 and less than 0.50: weak correlation.
• *r* greater than or equal to 0.50 and less than 0.70: moderate correlation.
• *r* greater than or equal to 0.70: strong correlation.

All analyses were conducted using SAS (version 9.4, SAS Institute, North Carolina State University).

## Results

### Respondent characteristics

**Fig. 2:**
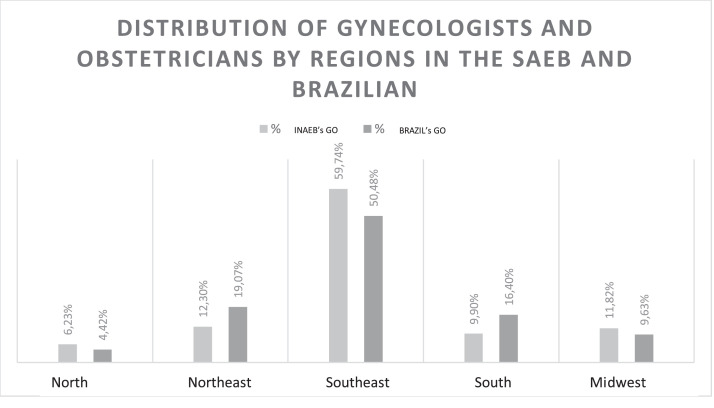
Distribution of participating gynecologists and obstetricians by geographic region of INAEB (n = 627) and Brazil (n = 37327), Ribeirão Preto, SP, Brazil.

Respondents of this study were located in five Brazilian regions and 25 states, including the Federal District (Fig. 2). No physicians in Amapá or Roraima responded. Physicians in the southeast region accounted for 60 % (n = 374) of the total respondents, including 41.54 % (n = 221) in São Paulo. Data with distribution proportionally similar to that of gynecologists and obstetricians in Brazil according to a document published by the Brazilian Medical Association of 2023 of Brazilian medical demography (p = 0.999) ^12^ (Fig. 2).

The mean respondent age was 42.9±13.8 years. The respondents from the central-west region were the youngest with a mean age of 39 years. A total of 78.95 % (n = 495) of respondents identified themselves as specialists, and 54.21 % (n = 322) had the title of Specialist in Gynecology and Obstetrics (*Título de Especialista em Ginecologia e Obstetrícia*). The mean time since graduation was 17.6±13.9 years (Table 1).

**Table 1:** Respondent characteristics (n = 627).

		Geographic distribution						
		Brazil	South	Southeast	Central-west	North	Northeast	*p*-value
**Age (mean in years ± SD)**		42.9 ± 13.80	44.02	43.27	38.95	43.32	43.74	
**Medical training time (Mean years ± SD)**		17.6 ± 13.9	19.02	17.95	13.62	17.59	18.60	
**Professional qualification**	Resident	126 (20.10%)		10 (16.13%)	82 (21.93%)	16 (21.62%)	7 (17.95%)	0.7533
	First year	22 (3.5%)		3 (30%)	13 (16.05%)	3 (18.75%)	1 (14.29%)	
	Second year	52 (8.3%)		6 (60%)	34 (41.98%)	5 (31.25%)	3 (42.86%)	
	Third year	47 (7.5%)		10 (10%)	32 (39.51%)	8 (50%)	3 (42.86%)	
	Subspecialty	3 (0.5%)		0	2 (2.47%)	0	0	
	Specialist	495 (78.95%)		51 (82.26%)	288 (77.01%)	58 (78.38%)	32 (82.05%)	
	Generalist	6 (0.96%)		1 (1.61%)	4 (1.07%)	0	0	
**Predominant area of activity**	Obstetrician	113 (22.83%)		12 (23.53%)	62 (21.53%)	14 (24.14%)	7 (21.88%)	0.2150
	Gynecologist	39 (7.88%)		3 (5.88%)	22 (7.64%)	11 (18.97%)	1 (3.13%)	
	G&O	333 (67.27%)		36 (70.59%)	198 (68.75%)	31 (53.45%)	24 (75%)	
	Mastologist	8 (1.62%)		0	4 (1.39%)	2 (3.45%)	0	
	Others	2 (0.40%)		0	2 (0.69%)	0	0	

Data are presented as number (frequency) or mean ± standard deviation.

SD, Standard Deviation.

### Characteristics of the infrastructure, resources, and workplaces

The majority of physicians worked primarily and exclusively in women's care and health services (50.8%, n = 318) and public services (62.3 %, n = 390) (p < 0.001) (Table 2).

**Table 2:** Type of institutions (n = 627).

Type of institution	Distribution						
	Brazil	South	Southeast	Central-west	North	Northeast	*p*-value
Public	221 (37.21%)	15 (24.59%)	104 (29.71%)	26 (36.62%)	24 (63.16%)^a^	51 (69.9%)^a^	<0.001^a^
Private	56 (9.43%)	2 (3.28%)	37 (10.57%)	9 (12.68%)	4 (10.53%)	4 (4.58%)	
Health insurance plan	41 (8.10%)	3 (4.92%)	40 (11.43%)	5 (7.04%	1 (2.63%)	2 (2.74%)	
Maternity ward in a public general hospital	169 (28.45%)	21 (34.43%)	110 (31.43%)	22 (30.99%)	6 (15.79%)	10 (13.7%)	
Maternity ward in a private general hospital	97 (16.33%)	20 (32.79%)^a^	59 (16.86%)	9 (12.68%)	3 (7.89%)	6 (8.22%)	

Data are presented as number (percentage).

a A significant difference was observed between the distribution of physicians in public maternity hospitals in the North and Northeast regions in relation to the other regions of the country and between the distribution of physicians who work in a maternity hospital located in a private general hospital in the southern region in relation to the other regions of the country.

Most respondents (86.76 %) reported access to an obstetric center, 81.98% to a clinical analysis laboratory, 80 % to essential laboratory tests for the diagnosis and follow-up of patients, and 65.39 % to a transfusion agency (Tables 3-5).

**Table 3:** Availability of structural resources (n = 627).

Structural resource	Availability						
	Brazil	South	Southeast	Central-west	North	Northeast	*p*-value
ER with a casualty room	504 (80.38%)	53 (85.48%)	297 (79.41%)	63 (85.14%)	31 (79.49%)	59 (76.62%)	0.5557
Obstetric center	544 (86.76%)	54 (87.1%)	322 (86.1%)	64 (86.49%)	35 (89.74%)	68 (88.31%)	0.9611
General adult ICU	418 (66.67%)	46 (74.19%)	272 (72.73%)	56 (75.68%)	21(53.85%)^a^	23(29.87%)^a^	<0.001^a^
Obstetric ICU	170 (27.11%)	10 (16.13%)	98 (26.20%)	18 (24.32%)	16 (41.03%)	27 (35.06%)	0.0322
Transfusion agency	410 (65.39%)	41 (66.13%)	250 (66.84%)	51 (68.92%)	25 (64.10%)	42 (54.55%)	0.3087
Clinical analysis lab	514 (81.98%)	51 (82.26%)	304 (81.28%)	68 (91.89%)	31 (79.49%)	59 (76.62%)	0.1528
Referral hospital	196 (31.26%)	24 (38.71%)	106 (28.34%)	27 (36.49%)	14 (35.9%)	24 (31.17%)	0.3522
Hospital transport	394 (62.84%)	39 (62.9%)	230 (61.5%)	51 (68.92%)	25 (64.1%)	49 (63.64%)	0.8255
No resources cited	5 (0.80%)	1 (1.61%)	3 (0.8%)	0	1 (2.56%)	0	0.5139

Data are presented as number (percentage).

ER, Emergency Room; lab, Laboratory; ICU, Intensive Care Unit.

a A significant difference was observed between the availability of a general adult ICU in the North and Northeast regions compared to the other regions of the country.

**Table 4:** Availability of laboratory tests (n = 627).

**Laboratory tests**	**Availability/percentage**						
	**Brazil**	**South**	**Southeast**	**Central-west**	**North**	**Northeast**	***p*-value**
Complete blood count	511(81.50%)	51 (82.26%)	303 (81.02%)	68 (91.89%)	31 (79.49%)	58 (75.32%)	0.1131
PT	500 (79.74%)	49 (79.03%)	302 (80.75%)	66 (89.19%)	30 (76.92%)	52 (67.53%)^a^	0.0204^a^
APTT	498 (79.43%)	49 (79.03%)	303 (81.02%)	66 (89.19%)	28 (71.79%)	51 (66.23%)	0.0059
Urea	511 (81.50%)	51 (82.26%)	301 (80.48%)	68 (91.89%)	31 (79.49%)	59 (76.62%)	0.1417
Creatinine	513 (81.82%)	51 (82.26%)	303 (81.02%)	68 (91.89%)	31 (79.49%)	59 (76.62%)	0.1498
Sodium	503 (80.22%)	51 (82.26%)	302 (80.75%)	66 (89.19%)	31 (79.49%)	52 (67.53%)^a^	0.0190^a^
Potassium	504 (80.38%)	51 (82.26%)	303 (81.02%)	66 (89.19%)	31 (79.49%)	52 (67.53%)^a^	0.0178^a^
Bilirubin	509 (81.18%)	51 (82.26%)	301 (80.48%)	67 (90.54%)	31 (79.49%)	59 (76.62%)	0.2351
GOT	511 (81.50%)	51 (82.26%)	301 (80.48%)	68 (91.89%)	31 (79.49%)	59 (76.62%)	0.1417
GPT	510 (81.34%)	51 (82.26%)	300 (80.21%)	68 (91.89%)	31 (79.49%)	59 (76.62%)	0.1368
LDH	504 (80.38%)	51 (82.26%)	301 (80.48%)	66 (89.19%)	30 (76.92%)	55 (71.43%)	0.0923
Arterial/venous blood gases	478 (76.24%)	51 (82.26%)	289 (77.27%)	62 (83.78%)	28 (71.79%)	47 (61.04%)^a^	0.0072^a^
Urine-1	511 (81.50%)	51 (82.26%)	301 (80.48%)	68 (91.89%)	31 (79.49%)	59 (76.62%)	0.1417
UPCR	423 (67.46%)	47 (75.81%)	260 (69.52%)	54 (71.97%)	24 (61.54%)^a^	37 (48.05%)^a^	0.0015^a^
lactate	458 (73.05%)	47 (75.81%)	284 (75.94%)	58 (78.38%)	26 (66.67%)^a^	42 (54.55%)^a^	0.0019^a^
C-reactive protein	501 (79.9%)	51 (82.26%)	299 (79.95%)	64 (86.49%)	31 (79.49%)	55 (71.43%)	0.2266

Data are presented as number (percentage).

PT, Prothrombin activity Time; APTT, Activated Partial Thromboplastin Time; GOT, Glutamic Oxaloacetic Transaminase; GPT, Glutamic Pyruvic Transaminase; LDH, Lactic Dehydrogenase; UPCR, Urine Protein to Creatinine Ratio.

a Significant statistical difference between data from the highlighted region and the other regions on the same line.

**Table 5:** Availability of resources (n = 627).

Resource		Brazil	South	Southeast	Central-west	North	Northeast	*p*-value
Puerperal hemorrhage	Oxytocin	594 (94.74%)	61 (98.39%)	350 (93.58%)	71 (95.95%)	38 (97.44%)	73 (94.81%)	0.4874
	Methergine	580 (92.50%)	60 (96.77%)	344 (91.98%)	70 (94.59%)	37 (94.87%)	68 (88.31%)	0.3418
	Tranexamic acid	580 (92.50%)	57 (91.94%)	345 (92.25%)	69 (93.24%)	36 (92.31%)	72 (93.51%)	0.9935
	Misoprostol	583 (92.98%)	59 (95.16%)	346 (92.51%)	68 (91.89%)	37 (94.87%)	72 (93.51%)	0.9163
	Intrauterine tamponade balloon	182 (30.74%)	26 (43.33%)	103 (29.43%)	22 (31.43%)	13 (34.21%)	18 (24.66%)	0.4443
	Hemostatic suture	383 (65.03%)	39 (63.93%)	224 (64.37%)	45 (64.29%)	30 (81.08%)	45 (62.5%)	0.0876
Hypertensive syndrome	Nifedipine	580 (92.50%)	60 (96.77%)	341 (91.18%)	67 (90.54%)	38 (97.44%)	73 (94.81%)	0.2934
	Hydralazine	585 (93.30%)	57 (91.94%)	345 (92.25%)	71 (95.95%)	38 (97.44%)	73 (94.81%)	0.5544
	Magnesium sulfate	588 (93.78%)	59 (95.16%)	349 (93.32%)	69 (93.24%)	37 (94.87%)	73 (94.81%)	0.9636
	Sodium nitroprusside	395 (63.00%)	35 (56.45%)	253 (67.65%)	46 (62.19%)	19 (48.72%)^a^	41 (53.25%)^a^	0.0247^a^
		19 (3.03%)	2 (3.23%)	8 (2.14%)	1 (1.35%)	3 (7.69%)	5 (6.49%)	0.1014
Septic shock	Broad-spectrum antibiotics	588 (93.78%)	59 (95.16%)	349 (93.32%)	71 (95.95%)	37 (94.87%)	71 (92.21%)	0.8545
	Vasopressors	579 (92.34%)	61 (98.39%)	342 (91.44%)	69 (93.24%)	36 (92.31%)	70 (90.91%)	0.4149
	Volume expanders	589 (93.94%)	60 (96.77%)	348 (93.05%)	70 (94.59%)	37 (94.87%)	73 (94.81%)	0.8068
	Blood components	536 (85.49%)	56 (90.32%)	323 (86.36%)	65 (87.84%)	34 (87.18%)	57 (74.03%)^a^	0.0411^a^
Abortion and GTD	Manual intrauterine aspiration	428 (68.26%)	38 (61.29%)	243 (64.97)	63 (85.14%)^a^	26 (66.67%)	57 (74.03%)	0.0067^a^
	Vacuum aspiration	130 (20.73%)	9 (14.52%)*	92 (24.6%)	8 (10.81%)	7 (17.95%)	14 (18.18%)	0.0445^a^
	Curettage	581 (92.66%)	60 (96.77%)	342 (91.44%)	70 (94.59%)	36 (92.31%)	72 (93.51%)	0.5834

Data are presented as number (percentage). Vasopressors include noradrenaline. Adrenaline.

GTD, Gestational Trophoblastic Disease.

a Significant statistical difference between data from the highlighted region and the other regions on the same line.

### Skills, safety, and knowledge of OE diagnosis and management

Nearly all of the respondents (93.47 %; n = 555) reported having provided care to a pregnant or postpartum patient with an obstetric emergency.

A total of 466 respondents (78.85 %) reported having knowledge regarding the diagnosis and management of OE, without statistically significant differences between the five Brazilian regions (p = 0.2184). A total of 386 respondents (61.41 %) reported undergoing specific preparatory training for the diagnosis and management of OE, including 55.74 % of respondents from the south, 67.71 % from the southeast, 57.75 % from the central-west, 68.42 % from the north, and 64.38 % from the northeast regions, without statistically significant differences between the five Brazilian regions (*p* = 0.5610).

Respondents from the southeast region participated in more OE courses conducted by the Associations of Gynecologists and Obstetricians of Minas Gerais (*Associação dos Ginecologistas e Obstetras de Minas ‒* SOGIMIG) and São Paulo (*Associação de Obstetrícia e Ginecologia do Estado de São ‒* SOGESP) than respondents from other regions (SOGIMIG: southeast, 11.76 %; south, 1.61 %; central-west, 1.35 %; north, 0 %; and northeast, 2.60 %; p *=* 0.0002; SOGESP: southeast, 23.26 %; south, 9.68 %; central-west, 4.05 %; north, 5.13 %; and northeast, 9.09 %; *p <* 0.001). In addition, 267 respondents (69.17 %) reported undergoing training during their residency, and 51 (13.2%) reported undergoing training during their fetal medicine specialization.

Of the 386 respondents who reported previous OE training, 322 (83.41 %) participated in training courses within the previous five years.

Access to periodic training strategies at their institution was reported by 228 respondents (36.36 %), including 42.82 % of respondents from the southeast region and 51.35% from the north regions (p = 0.0128).

### Protocols, processes, and team organization

A total of 471 respondents (79.43 %) reported that the patient's first appointment was with an obstetrician (Table 6).

**Table 6:** Characteristics of protocols. processes. and team organization (n = 593).

	Brazil	South	Southeast	Central-west	North	Northeast	*p*-value
**First appointment with**							0.2492
Clinician	30 (5.06%)	3 (5%)	17 (4.87%)	4 (5.8%)	5(13%)	1 (1.4%)	
Emergency physician	10 (1.69%)	2 (3.2%)	7 (0.2%)	1 (1.4%)	0	0	
Family physician	9 (1.52%)	1 (1.6%)	5 (0.1%)	1 (1.4%)	0	2 (2.8%)	
Obstetrician	471 (79.43%)	52 (85.2%)	280 (80.2%)	56 (78.8%)	27 (71%)	55 (75.3%)	
Non-physician (nurse/nursing technician)	73 (12.31%)	3 (5%)	40 (14.6%)	9 (12.6%)	6 (16%)	15 (20.5%)	
**Risk classification implementation**							0.0610
Yes	440 (74.38%)	42 (68.85%)	266 (76.22%)	46 (65.71%)	29 (76.32%)	56 (76.71%)	
No	107 (18.04%)	14 (22.95%)	59 (16.91%)	12 (17.14%)	9 (23.68%)	13 (17.81%)	
Unknown	46 (7.58%)	5 (8.20%)	24 (6.88%)	12 (17.14%)	0	4 (5.48%)	
**MEOWS implementation**							**0.0032**^a^
Yes	201 (33.09%)	16 (26.23%)	138 (38.54%)	12 (16.9%)^a^	17 (44.74%)	17 (23.29%)	
No	257 (43.34%)	31 (50.82%)	134 (38.40%)	40 (56.34%)	16 (42.11%)	36 (49.32%)	
Unknown	135 (22.77%)	14 (22.95%)	77 (22.06%)	19 (26.76%)	5 (13.16%)^a^	20 (27.40%)	
**Quick response team**							0.0098^a^
Yes	276 (46.54%)	28 (45.9%)	179 (51.14%)	22 (30.99%)^a^	23 (60.53%)	24 (33.33%)^a^	
No	274 (46.21%)	30 (49.18%)	144 (41.14%)^a^	42 (59.15%)	14 (36.84%)^a^	43 (59.72%)	
Unknown	43 (7.25%)	3 (4.92%)	27 (7.71%)	7 (9.86%)	1 (2.63%)	5 (6.94%)	
**Emergency boxes and/or carts**							0.394
Yes	425 (71.68%)	46 (75.41%)	264 (76.08%)	45 (64.29%)	29 (78.38%)	40 (55.56%)	
No	101 (17.03%)	9 (14.75%)	50 (14.41%)	16 (22.86%)	5 (13.51%)	21 (29.17%)	
Unknown	67 (11.29%)	6 (9.84%)	33 (9.51%)	9 (12.86%)	3 (8.11%)	11 (15.28%)	

Data are presented as number (percentage).

MEOWS, Modified Early Obstetric Warning Score.

a Significant statistical difference between the highlighted region data and the other regions in the same line.

A risk classification protocol was implemented in the emergency room at the institutions of 440 respondents (74.38 %). A total of 201 (33.09 %) respondents reported that the MEOWS was used at their institution, including 38.54 % in the southeast region and 44.74 % in the north region (p = 0.0032). The MEOWS protocol was used by 16.9 % of respondents in the central-west region. A total of 7.58% of respondents were unaware of the risk classification protocol, and 22.77% were unaware of the obstetric risk classification system (Table 6).

A quick response team is available for emergencies at the institutions of 276 (46.54 %) respondents, and 425 (71.68 %) respondents reported that they work in services where there is a box and/or cart assembly protocol for OE, including 55.26 % of respondents in the northeast region (Table 6).

### Exploratory analysis of correlations

Based on the direct (bleeding, abortion, puerperal infection, and hypertensive syndromes) and indirect MMRs calculated using consolidated data published in the 2020 Mortality Information System of the Ministry of Health, lower availability of a rapid response team was associated with a greater MM from indirect causes (*r* = 0.9000, *p* = 0.0374) (Table 7).

**Table 7:** Correlations between prevalence data on resource availability. training courses. and organization of health services with prevalence of maternal deaths

Correlated data	Spearman's correlation (*r*)	*p*-value
Attended a specific OE course × prevalence of maternal deaths from direct obstetric causes	-0.56429	0.3217
Attended a specific OE course × prevalence of maternal deaths from indirect obstetric causes	0.23684	0.7013
Attended some OE training × prevalence of maternal deaths from direct obstetric causes	-0.5000	0.3910
Attended some OE training × prevalence of maternal deaths from indirect obstetric causes	0.20520	0.7406
Periodic training at the institution where they work × prevalence of maternal deaths from direct obstetric causes	-0.56429	0.3217
Periodic training at the institution where they work × prevalence of maternal deaths from indirect obstetric causes	0.02632	0.9665
Has a risk protocol implemented × prevalence of maternal deaths from direct obstetric causes	-0.5000	0.3910
Has a risk protocol implemented × prevalence of maternal deaths from indirect obstetric causes	-0.80000	0.1041
MEOWS protocol implemented × prevalence of maternal deaths from direct obstetric causes	-0.10260	0.8696
MEOWS protocol implemented × prevalence of maternal deaths from indirect obstetric causes	-0.80000	0.1041
Rapid response team × prevalence of maternal deaths from direct obstetric causes	0.10260	0.8696
**Rapid response team × prevalence of maternal deaths from indirect obstetric causes**	**-0.90000**	**0.0374**^a^
Presence of a protocol for assembling boxes or carts × prevalence of maternal deaths from direct obstetric causes	0.41039	0.4925
Presence of a protocol for assembling boxes or carts × prevalence of maternal deaths from indirect obstetric causes	0.67082	0.2152
Opportunity to perform a hemostatic uterine suture technique × prevalence of maternal deaths from hemorrhage	0.46169	0.4338
Presence of protocol for assembling boxes or carts × prevalence of maternal deaths from abortion	0.30000	0.6238
Presence of a protocol for assembling boxes or carts × prevalence of maternal deaths from hemorrhage	0.60000	0.2848
Presence of a protocol for assembling boxes or carts × prevalence of maternal deaths from hypertensive syndromes	-0.50000	0.3910
Presence of protocol for assembling boxes or carts × prevalence of maternal deaths from puerperal infection	0.35355	0.5594
Opportunity to perform a hemostatic uterine suture technique × prevalence of maternal deaths from hemorrhage	-0.30000	0.6238
Use of intrauterine balloon tamponade × prevalence of maternal deaths from hemorrhage	0.60000	0.2848
Availability of oxytocin × prevalence of maternal deaths from hemorrhage	0.50000	0.3910
Availability of methergine × prevalence of maternal deaths from hemorrhage	0.66689	0.2189
Availability of tranexamic acid × prevalence of maternal deaths from hemorrhage	-0.22361	0.7177
Availability of misoprostol × prevalence of maternal deaths from hemorrhage	0.15811	0.7995
Availability of nifedipine × prevalence of maternal deaths from hypertensive syndromes	0.63246	0.2522
Availability of hydralazine × prevalence of maternal deaths from hypertensive syndromes	0.05130	0.9347
Availability of magnesium sulfate × prevalence of maternal deaths from hypertensive syndromes	-0.86603	0.0577
Availability of sodium nitroprusside × prevalence of maternal deaths from hypertensive syndromes	-0.70000	0.1881
Availability of broad-spectrum antibiotics × prevalence of maternal deaths from puerperal infection	-0.72548	0.1654
Availability of vasopressors × prevalence of maternal deaths from puerperal infection	-0.36274	0.5485
Availability of volume expanders × prevalence of maternal deaths from puerperal infection	0.00000	1.0000
Availability of blood components × prevalence of maternal deaths from puerperal infection	-0.35355	0.5594
Availability of blood components × prevalence of maternal deaths from hemorrhage	0.70000	0.1881
Availability of MVA × prevalence of maternal deaths from abortion	0.20520	0.7406
Availability of vacuum aspiration × prevalence of maternal deaths from abortion	0.65789	0.2275
Availability of curette × prevalence of maternal deaths from abortion	-0.82078	0.0886
Rapid response team × prevalence of maternal deaths from abortion	0.61559	0.2690
Rapid response team × prevalence of maternal deaths from hemorrhage	-0.10000	0.8729
Rapid response team × prevalence of maternal deaths from puerperal infection	0.70711	0.1817
Rapid response team × prevalence of maternal deaths from hypertensive syndromes	-0.20000	0.7471
Ability to manage COVID-19 patients × prevalence of maternal deaths from indirect causes	-0.26316	0.6688
Received a training course or underwent training on managing COVID-19 patients × prevalence of maternal deaths from indirect causes	-0.34412	0.5707

OE, Obstetric Emergencies; MEOWS, Modified Early Obstetric Warning Score; MVA, Manual Vacuum Aspiration; COVID-19: Coronavirus Disease 2019.

*r*≥0.30: weak correlation; *r*≥0.50: moderate correlation; *r*≥0.70: strong correlation.

a Significant statistical association.

## Discussion

The results of this study are derived from the reports of physicians who work in gynecology and obstetrics in different socioeconomic and cultural settings and are therefore directly influenced by the physicians’ specific patient populations.

Brazil is a country of continental dimensions, with five macro-regions characterized by distinct cultural and socioeconomic realities and an uneven population distribution. Data published by the Brazilian Institute of Geography and Statistics (IBGE) estimate that in 2021 the Brazilian population corresponded to 213.3 million inhabitants, with the Southeast region concentrating 42.2 % of the population.^13^ The Southeast is not only the most populous region in the country but also has the highest municipal Human Development Index (HDI, which considers longevity, educational level, and income) (0.766), with 55.2 % of the Product Brazilian Gross Domestic (GDP).^14^

A greater representation of physicians from the Southeast 60 % (n = 374) of the total number of respondents. Considering that the data in the present study are predominantly derived from physicians from the Southeast region (60 %), which corresponds to the richest economy in the country, which has greater availability of health resources, the regional reality directly impacts the results observed in this study at the national level.^14^

The availability of adult Intensive Care Unit (ICU) beds was greater in the south, southeast, and central-west regions than in the north and northeast regions (Table 3). However, the number of adult ICU beds exclusively dedicated to obstetric conditions was low in all of the regions. The availabilities of essential tests for identifying severe conditions and diagnosing and managing hypertensive syndromes and sepsis were higher in the south, southeast, and central-west regions than in the north and northeast regions (Table 4).

The availability of medications broadly used in clinical practice for the main causes of OE was adequate in approximately 90% of the respondents’ institutions. However, instruments, devices, and medications used to treat more complex and severe conditions that are generally refractory to initial treatments, such as intrauterine tamponade balloon, vacuum aspirator, sodium nitroprusside, and Manual Vacuum Aspiration (MVA) had lower availability (Table 5).

The MVA was less available than uterine curettage, which is in contrast to the recommendations by the WHO and FEBRASGO. According to the WHO, MVA is the first choice for the safe abortion of pregnancies of less than 12‒14 weeks. This instrument is highly effective and requires only 3–10 minutes to complete an abortion with low complication rates, including blood loss and uterine perforation. A greater dissemination of the use of MVA is necessary to reduce the MMR.^15^^,^^16^

The intrauterine balloon, which is not widely accessible in Brazil, is an extremely important tool in patients with puerperal hemorrhage refractory to pharmacological therapy, with a success rate of 80 % and low morbidity and mortality that allows for the avoidance of surgical interventions and the preservation of the reproductive future of the patient.^17^

Early diagnoses and treatments of patients with severe conditions are essential for adequate care of obstetric patients, which requires the organization of care processes and the implementation of risk classification and the MEOWS.^18^ Low rates of obstetric risk classification implementation were observed in all Brazilian regions in this study.

In the UK, the MEOWS is implemented at 100 % of institutions as an effective strategy for reducing maternal morbidity and mortality when applied by trained professionals.^19^^,^^20^ The use of this obstetric screening tool must be accompanied by specific training to be effective.^21^^,^^22^ In this study, a significant portion of respondents were unaware of the MEOWS.

The respondents in this study reported having the confidence and knowledge to care for pregnant women and mothers under severe MM conditions. In contrast, few respondents reported access to periodic training, though the training was more available in the southeast and northern regions.

Continuing education is an important tool for the reduction of MM. Several studies conducted in low-income countries have demonstrated that periodic training of a multidisciplinary team and OE training for physicians, regardless of the full availability or scarcity of structural resources (such as medications and tests), can significantly reduce the MM.^23^, ^24^, ^25^, ^26^, ^27^

Increasing the efficiency of obstetric care can effectively reduce health costs.^28^ In Kenya, for every $1 invested in periodic OE training courses, the social impact was equivalent to 12.74 dollars.^29^

Advanced Life Support in Obstetrics (ALSO) was created in 1991 to improve physicians’ skills related to OE and significantly affected the MMR. A 2017 prospective study conducted in low-income countries such as Guatemala, Honduras, Colombia, and Tanzania reported a reduction in the MMR after ALSO was implemented by the local medical team, reflecting the acquisition of practical skills and confidence by professionals. Therefore, obstetricians should be trained to save lives even in areas with few resources.^27^

Training for the care of the most common peripartum complications in hospitals in Kenya reduced the number of patients with puerperal hemorrhage.^30^ In obstetric and neonatal care services in Senegal and Mali, which implemented a systematic review of maternal deaths and best health practices, the MM was significantly reduced compared to that in hospitals where such practices were not implemented.^26^

Therefore, the MMR can be reduced via the dissemination of OE training courses. Internal training was more accessible in the north region in this study, which may be associated with greater completion rates of the Zero Maternal Death course in this region. This course was developed by the Ministry of Health in partnership with the Pan American Health Organization/WHO to prevent MM from preventable hemorrhagic causes and accelerate the reduction of the Brazilian MMR by training professionals in practical work and organizing health processes.^31^^,^^32^

Another important tool already implemented in the southeast region is theoretical-practical courses regarding OE at gynecology and obstetrician societies. The latter was structured by gynecologists and obstetricians to decrease the MMR in São Paulo through the implementation of training protocols, with theoretical and practical activities being developed to systematize the care of severe MM conditions (from diagnosis to treatment).

Training the care team, encouraging the implementation of standardized protocols, and stimulating care flow and institutional structure changes are the pillars of this course and essential factors in reducing the MMR.^28^^,^^33^

## Conclusions

A high MMR may be related to deficient health service organization, low implementation of risk classification and management protocols regarding severe maternal and fetal conditions, and lack of access to continued and specific training programs by the medical team.

Factors that could be improved to reduce the Brazilian MMR to less than 35 maternal deaths per 100,000 live births by 2030 must be identified, including supporting the implementation of OE education and training sessions.

Several factors affect the Brazilian MMR. According to obstetricians, Brazilian institutions have care teams, essential medications, obstetric centers, and clinical analysis laboratories, but lack systematized processes and permanent professional training for the qualified care of OE, corroborating the increase of rapid response teams, which can impact the reduction of maternal mortality.

## Declaration of competing interest

The authors declare no conflicts of interest.
